# The metabolic debt model: cumulative bioenergetic burden and network vulnerability in brain disorders—a hypothesis article

**DOI:** 10.1093/braincomms/fcag359

**Published:** 2026-10-16

**Authors:** Effiong N Effiong, Daniel M Gimbason, Yakubu B Ngwai, Julius B Gajere

**Affiliations:** Global Health and Infectious Diseases Control Institute, Nasarawa State University, Keffi 961101, Nigeria; Center for Applied Health Research and Development, Efficacy International School, Kaduna 800100, Nigeria; Global Health and Infectious Diseases Control Institute, Nasarawa State University, Keffi 961101, Nigeria; Global Health and Infectious Diseases Control Institute, Nasarawa State University, Keffi 961101, Nigeria; Community AIDS/HIV and Tuberculosis Foundation, Kaduna 800100, Nigeria

## Abstract

Effiong *et al*. propose the Metabolic Debt Model, a hypothesis-generating framework that links four metabolic constraints, phosphocreatine buffering, astrocyte–neuron coupling, mitochondrial flexibility and nicotinamide adenine dinucleotide^+^–dependent recovery to network-level dysfunction and clinical symptoms in myalgic encephalomyelitis and long coronavirus diseases.

***The human brain operates under high energetic demand. Fatigue, cognitive slowing and post-exertional symptom exacerbation are shared features across occupational burnout, myalgic encephalomyelitis/chronic fatigue syndrome and post-acute sequelae of severe acute respiratory syndrome coronavirus 2 infection. Despite their expanding population burden, these conditions remain mechanistically fragmented.***

## Introduction: the need for a unifying framework

The brain is an energetically constrained organ whose functional integrity depends on tightly regulated metabolic supply–demand coupling.^1,2^ Synaptic transmission, action potential propagation and plasticity require continuous ATP production, largely dependent on mitochondrial oxidative phosphorylation.^3^ Constituting only ∼2% of body mass, the brain consumes ∼20% of resting energy expenditure and relies on tightly coupled metabolic processes with minimal intrinsic buffering capacity.^1,2^

Fatigue-related disorders including occupational burnout, myalgic encephalomyelitis/chronic fatigue syndrome (ME/CFS) and long coronavirus disease (COVID) share a common phenomenology despite their distinct clinical labels: abrupt collapse following effort, delayed symptom exacerbation hours to days later and prolonged recovery trajectories.^4-6^ Long COVID, in particular, has been associated with persistent metabolic and immune dysregulation that may intersect with the neuroenergetic mechanisms proposed here.^7^ These conditions affect millions worldwide and represent a substantial and growing burden on health systems, yet their pathophysiology remains poorly understood.

### Rationale for cross-condition synthesis

The authors explicitly note that ME/CFS, long COVID and occupational burnout have distinct aetiologies, biomarker profiles and clinical trajectories. For example, post-viral fatigue syndromes show persistent immune dysregulation not consistently observed in stress-related burnout. The Metabolic Debt Model (MDM) does not propose identical mechanisms across conditions. Instead, the model hypothesizes that diverse triggers, including viral infection, chronic stress, and metabolic insult, may converge on a final common pathway of neuroenergetic constraint in susceptible individuals. The model is intended to apply to subsets of patients within each diagnostic category, not uniformly across all affected individuals.

This raises a broader question: can mechanisms observed in neurodegenerative diseases inform our understanding of fatigue-spectrum disorders? Parallel insights from neurodegenerative disease research have reframed Alzheimer’s disease as a disorder of metabolic inflexibility, in which adaptive cellular metabolic shifts become pathologically rigid and drive disease progression.^8^ This biphasic trajectory from early, glia-driven hypermetabolism to late-stage hypometabolism and energy collapse offers a conceptual template for understanding how initially compensatory metabolic adaptations may become maladaptive in fatigue-spectrum disorders.

Contemporary neuroscience has increasingly recognized that metabolic dysfunction may underlie a range of neurological and psychiatric conditions. Fatigue is a prominent and disabling symptom in multiple sclerosis, Parkinson’s disease, traumatic brain injury and post-stroke syndromes, yet its mechanisms remain obscure. A framework that integrates cellular energetics with systems-level neural function could have broad translational relevance. While the MDM may have broader applicability, this article synthesizes evidence primarily from ME/CFS and long COVID, where the neuroenergetic evidence base is most developed.

This Hypothesis article proposes the MDM as a hypothesis-generating framework to integrate diverse observations and guide future investigation across fatigue-related conditions. Rather than asserting established mechanisms, the MDM offers a coherent architecture for generating testable predictions about the role of neuroenergetic dysfunction in fatigue, with implications for understanding fatigue in both primary neurological disorders and post-viral syndromes.

## The MDM: core premise

The MDM proposes that fatigue-spectrum disorders may arise from impaired energetic solvency in high-demand neural systems, a chronic imbalance between energy supply and demand that constrains cognitive and physical performance. The model identifies four interrelated metabolic processes that may contribute to this imbalance, presented here as plausible pathways requiring empirical validation rather than established mechanisms. These processes are conceptualized as interacting constraints within a multi-scale neuroenergetic system, where dysfunction at one level may propagate to affect system-wide function.

## Four pillars of the MDM

Key technical terms and acronyms used in the following sections are defined in Table 1.

**Table 1:** Acronym definitions

Acronym	Definition
ADP	Adenosine diphosphate
AMPK	AMP-activated protein kinase
ANLS	Astrocyte–neuron lactate shuttle
ATP	Adenosine triphosphate
Cr	Creatine (unphosphorylated form of PCr)
CSF	Cerebrospinal fluid
DMN	Default mode network
EEG	Electroencephalography
fMRI	Functional MRI
GFAP	Glial fibrillary acidic protein
MDM	Metabolic Debt Model
ME/CFS	Myalgic encephalomyelitis/chronic fatigue syndrome
MRS	Magnetic resonance spectroscopy
NAD^+^	Nicotinamide adenine dinucleotide (oxidized form)
NADH	Nicotinamide adenine dinucleotide (reduced form)
NfL	Neurofilament light chain
NMetNet	Neural metabolic network
OXPHOS	Oxidative phosphorylation
PBMC	Peripheral blood mononuclear cell
PCr	Phosphocreatine
τPCr	Phosphocreatine recovery time constant
PEM	Post-exertional malaise
RNA	Ribonucleic acid
^31^P-MRS	Phosphorus-31 magnetic resonance spectroscopy
^1^H-MRS	Proton magnetic resonance spectroscopy

## Pillar I: phosphocreatine buffering failure

### Hypothesized mechanism

Neural computation depends on continuous ATP availability. We hypothesize that in fatigue-spectrum disorders, this buffering capacity may be compromised, leading to premature depletion and delayed recovery. The phosphocreatine (PCr) system acts as an ultra-rapid energy buffer, stabilizing ATP levels during high demand by donating a phosphate group to regenerate ATP from adenosine diphosphate (ADP).^3,9^ This high-energy phosphate reservoir enables neurons to sustain firing rates and oscillatory dynamics essential for attention, working memory and conscious processing during transient increases in metabolic demand.

### Evidence consistent with this hypothesis

In vivo magnetic resonance spectroscopy studies have reported prolonged post-activation recovery kinetics in ME/CFS and long COVID, suggesting impaired energetic buffering capacity.^10,11^ Kato *et al*.^10^ used phosphorus-31 magnetic resonance spectroscopy (^31^P-MRS) to demonstrate reduced PCr/ATP ratios in mental fatigue, while Godlewska *et al*.^11^ used 7T MRS to identify metabolic abnormalities in ME/CFS and long COVID consistent with bioenergetic dysfunction. These findings are particularly notable in high-demand cortical regions including prefrontal and parietal areas that support executive control.^12^ One interpretation of these findings is impaired energetic buffering though causal relationships remain to be established. Figure 1 illustrates the hypothesized slow-recharge phenotype derived from these observations.^10,11^

**Figure 1:**
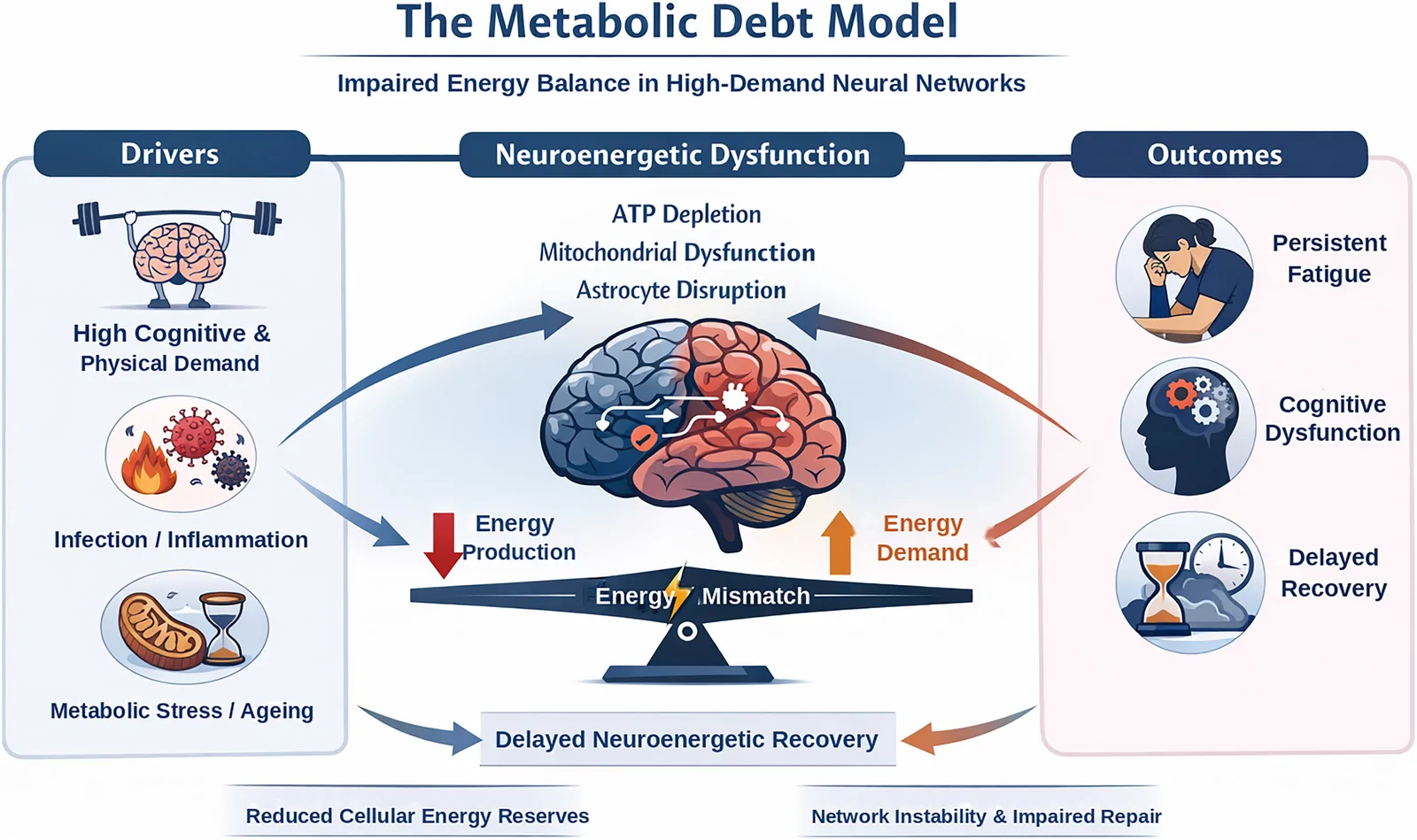
**The slow-recharge phenotype of PCr recovery.** Schematic of hypothesized PCr recovery dynamics based on published data from Kato *et al*.^10^ and Godlewska *et al*.^11^., Representative recovery time constants are shown: τPCr ∼30 s in healthy individuals versus ∼90 s in the hypothesized prolonged PCr recovery phenotype. Shaded regions indicate between-subject variability. The inset shows the creatine kinase reaction (PCr + ADP → Cr + ATP). This schematic represents a testable prediction of the MDM, not original experimental data.

### Hypothesis to test

PCr recovery kinetics (τPCr) will correlate with symptom severity and predict post-exertional relapse more strongly than static metabolic markers.^10,11^ This prediction can be tested through longitudinal ^31^P-MRS studies incorporating cognitive or physical challenge paradigms.

## Pillar II: astrocyte–neuron metabolic uncoupling

### Hypothesized mechanism

Sustained cognitive processing depends on the astrocyte–neuron lactate shuttle (ANLS). We hypothesize that disruption of this metabolic partnership may contribute to the subjective experience of cognitive slowing and mental fatigue.^13-15^ This coupling mechanism is particularly important during prolonged attention, memory retrieval and network integration, where neuronal energy demand remains elevated over minutes rather than seconds. A recent comprehensive review emphasizes that neurons and astrocytes operate as a metabolically coupled unit optimized to sustain the energetic demands of neurotransmission while ensuring neuroprotection.^16^ Disruption of this neuron–astrocyte metabolic unit contributes to numerous nervous system diseases, underscoring the critical importance of intercellular metabolic cooperation for brain function. Disruption of this metabolic partnership may contribute to the subjective experience of cognitive slowing and mental fatigue commonly described as brain fog.

### Evidence consistent with this hypothesis

Recent work has identified astrocyte-derived lactate as a critical mediator of cognitive resilience under stress, directly linking glial metabolic function to stress-related neuropsychiatric phenotypes.^17^ Magnetic resonance spectroscopy studies report elevated cortical and ventricular lactate in ME/CFS and long COVID, potentially reflecting inefficient metabolic handoff between glial and neuronal compartments.^18,19^ However, direct evidence linking ANLS dysfunction to brain fog in humans remains limited, and the interpretation of elevated lactate remains debated.

Metabolic profiling studies have further demonstrated that the neurometabolic network conditional dependencies between metabolites including creatine, glutathione and N-acetyl aspartate show altered connectivity in functional neurological disorder, suggesting bioenergetic dysregulation and increased oxidative stress vulnerability.^20^ These findings indicate that metabolic network architecture may be disrupted across multiple conditions characterized by fatigue and functional impairment.

### Hypothesis to test

Elevated cortical lactate on proton magnetic resonance spectroscopy (^1^H-MRS) will correlate with cognitive performance measures and symptom severity, particularly during cognitively demanding tasks. This association, if confirmed, would support the hypothesis that metabolic coupling failure contributes to cognitive symptoms.

## Pillar III: mitochondrial flexibility collapse

### Hypothesized mechanism

Sustained neural function depends on mitochondrial metabolic flexibility. We hypothesize that in fatigue-spectrum disorders, this adaptive capacity may be constrained to dynamically adjust substrate utilization and respiratory output in response to fluctuating energetic demands.^21^ This adaptability is essential for maintaining neural stability during prolonged cognitive engagement, stress or recovery from exertion. In fatigue-spectrum disorders, this adaptive capacity may be constrained, leading to an inability to match energy supply with sustained demand.

### Evidence

The concept of mitochondrial psychobiology proposes that mitochondria may play roles in neural resilience and stress adaptation, though direct human evidence remains preliminary and largely indirect.^22^ As Picard *et al.*^22^ note, rigorously testing this hypothesis in humans will require methods informed by principles of mitochondrial biology. The framework outlines plausible pathways whose strongest support derives from cellular and animal studies.

Lien *et al.*^23^ documented abnormal lactate responses to repeated exercise in ME/CFS, which may reflect broader metabolic dysregulation, though direct measures of oxidative phosphorylation were not assessed. More recently, Keller *et al.*^24^ demonstrated reduced oxygen consumption and abnormal metabolic responses during 2-day cardiopulmonary exercise testing in ME/CFS, providing further evidence of impaired metabolic flexibility. Vu *et al.*^25^ identified immune dysregulation in ME/CFS, including monocyte abnormalities and downregulation of ribosomal protein genes, but did not report coordinated downregulation of mitochondrial gene expression or oxidative phosphorylation (OXPHOS) pathways. Metabolomic studies have further identified distinct metabolic features in ME/CFS, including abnormalities in sphingolipid and phospholipid metabolism that may intersect with mitochondrial function.^26^ However, direct evidence linking peripheral mitochondrial measures to brain energetics is lacking.

### Hypothesis to test

Mitochondrial respiratory capacity measured via peripheral blood mononuclear cell (PBMC) respirometry will correlate with symptom severity and predict post-exertional malaise following standardized exercise challenge. This prediction can be tested through longitudinal studies incorporating physiological challenge paradigms.

## Pillar IV: impaired metabolic debt repayment

### Hypothesized mechanism

In healthy systems, metabolic by-products accumulated during wakefulness are cleared during sleep through two interdependent mechanisms: synaptic renormalization and glymphatic clearance.^27,28^ We hypothesize that failure of these recovery mechanisms may contribute to symptom persistence and unrefreshing sleep. These processes are regulated by cellular energy sensors, including nicotinamide adenine dinucleotide^+^ (NAD^+^)–dependent sirtuins and adenosine monophosphate (AMP)-activated protein kinase.^29,30^ Failure of these repayment mechanisms may contribute to symptom persistence and the experience of unrefreshing sleep.

### Evidence

Chronic stressors and neuroinflammation may disrupt these pathways, though direct evidence in fatigue-spectrum disorders is limited. NAD^+^ depletion has been observed in inflammatory conditions, and glymphatic dysfunction has been implicated in various neurological disorders.^31^ Sleep disruption is a near-universal feature of fatigue-spectrum disorders, and its relationship to metabolic recovery warrants systematic investigation.

### Hypothesis to test

Measures of NAD^+^ status and glymphatic function will correlate with sleep quality and symptom severity. This hypothesis could be tested through studies combining blood-based NAD^+^ metabolomics, sleep electroencephalography and emerging MRI-based measures of glymphatic function.

## Network-level consequences of neuroenergetic constraint

### From cellular constraints to network dysfunction

Cellular metabolic constraints may propagate upward to affect large-scale brain networks. Large-scale brain networks operate within a hierarchy of energetic preservation, whereby high-cost integrative functions are preferentially downregulated under conditions of metabolic stress.^32^ The salience network, which mediates switching between internally focused default mode network and externally directed executive networks, is energetically demanding and may be particularly vulnerable to metabolic constraint.^33^ Disruption within thalamocortical circuits, which play central roles in sensory gating and rhythmic coordination, may further contribute to sensory hypersensitivity and cognitive overload.

A transdiagnostic meta-analysis by Schumann *et al.*^34^ identified a widespread cortical-subcortical fatigue network involving frontal, limbic, basal ganglia and parietal structures, with key nodes in the lateral frontal cortex, cingulate cortex, insula, thalamus, precuneus and caudate. These findings support the theory of thalamic–striatal–cortical dysfunction, which may impair compensatory mechanisms related to mental fatigue. High-degree network hubs, including default mode network nodes and prefrontal cortex, have elevated metabolic demands and may be preferentially vulnerable to energetic constraint.^35^ This ‘hub vulnerability’ hypothesis predicts that metabolic debt should disproportionately affect global network efficiency and small-world properties.

### Evidence from neuroimaging

Neuroimaging studies in long COVID and ME/CFS have reported reproducible alterations in core cognitive networks, including hypometabolism within the salience network and instability of the default mode network.^18,36,37^ These patterns are consistent with adaptive downregulation of high-cost neural activity under conditions of energetic constraint, though direct evidence linking network-level changes to cellular metabolic measures remains limited.

### Graded vulnerability across the burden spectrum

The MDM conceptualizes metabolic burden as producing graded effects on network function:

- Mild metabolic burden → subtle cognitive slowing, reduced network efficiency during high-demand tasks.
- Moderate burden → affective dysregulation, altered salience network dynamics.
- Severe burden → structural and connectivity alterations, persistent network instability.

This dimensional framing aligns with contemporary approaches in systems neuroscience and positions fatigue-spectrum disorders along a continuum of neuroenergetic impairment rather than as categorical entities.

#### Hypothesis to test

Network-level dysfunction measured via resting-state and task-based functional MRI will correlate with cellular metabolic markers (PCr recovery kinetics, lactate levels, mitochondrial function) and symptom severity. This multi-scale prediction requires studies combining neuroimaging with peripheral and central metabolic measures. Figure 2 provides a visual summary of the complete MDM, illustrating how the four pillars interconnect with upstream stressors, network-level consequences and translational pathways.

**Figure 2:**
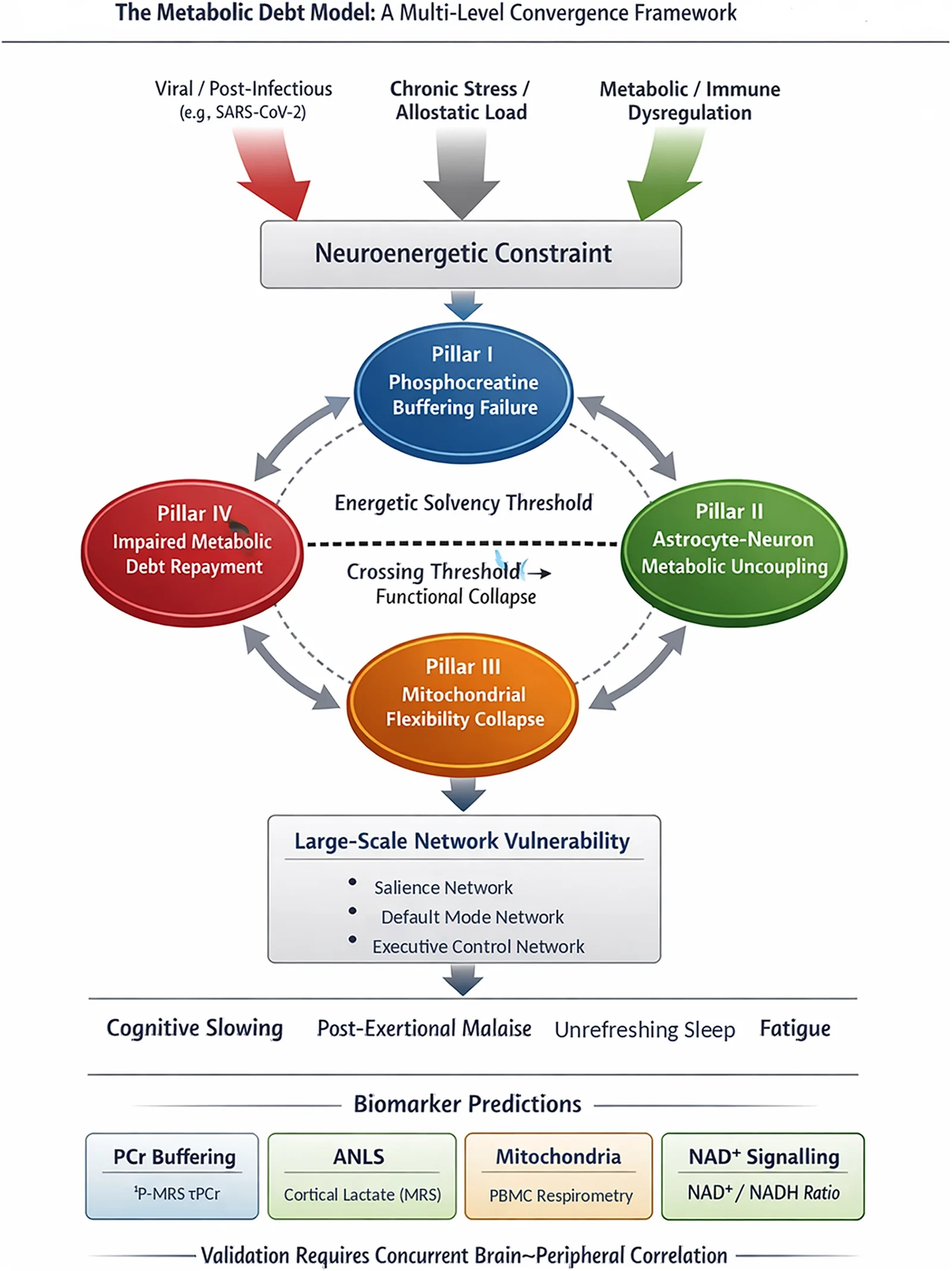
**The Metabolic Debt Model: a systems-level framework.** (**A**) Four interrelated metabolic pathways hypothesized to contribute to neuroenergetic constraint. (**B**) Diverse upstream stressors, including viral, stress-related, and metabolic factors, may converge on these pathways. (**C**) Cellular constraints may propagate to large-scale network dysfunction, including salience network hypometabolism and default mode network instability, with hub nodes exhibiting preferential vulnerability. (**D**) The model generates testable predictions for clinical assessment and intervention. This framework is presented as a hypothesis-generating model requiring empirical validation.

### Translational pathways: towards testable biomarkers

For readers unfamiliar with the technical terminology used throughout this article, Table 1 provides definitions of all acronyms. The MDM’s primary value lies in generating testable hypotheses. Table 2 outlines candidate biomarkers for each pillar, with the critical caveat that their relationship to brain energetics requires validation. For readers seeking definitions of technical terms, a Glossary of Key Terms is available in the Supplementary material.

**Table 2:** Candidate biomarkers for testing the MDM

Domain of energetic constraint	Candidate biomarker	Primary research question	Key methodological considerations
Energetic buffering	^31^P-MRS PCr recovery kinetics (τPCr)	Does delayed PCr recovery following cognitive or physical challenge predict post-exertional symptom exacerbation?	Requires high-field MRS; technically demanding; limited global availability
Metabolic coupling	Cortical or ventricular lactate (^1^H-MRS)	Does elevated lactate correlate with cognitive impairment and subjective fatigue severity?	Lactate elevation does not directly confirm astrocyte–neuron shuttle dysfunction
Mitochondrial flexibility	PBMC respirometry (oxygen consumption rate; spare respiratory capacity)	Does peripheral mitochondrial respiratory capacity predict relapse severity after exertion?	Peripheral measures may not accurately reflect central nervous system energetics
Recovery signaling	Blood NAD^+^/NADH ratio	Does NAD^+^ status predict sleep quality, recovery efficiency, or therapeutic response?	Peripheral NAD^+^ may not mirror brain concentrations; dynamic sampling required

Concurrent validation with central neuroimaging measures is required before peripheral biomarkers can be considered proxies for brain energetics. Peripheral biomarkers such as PBMC respirometry and blood NAD^+^ may reflect systemic processes rather than brain-specific energetics. Demonstrating correlations with brain measures (e.g. via MRS) is essential for validating their use as proxies.

A key translational priority is identifying relationships between advanced neuroimaging biomarkers and accessible plasma measures. Emerging evidence demonstrates that serum neurofilament light chain (NfL) and glial fibrillary acidic protein markers of neuroaxonal injury and astrocytic activation are elevated in post-COVID patients and correlate with cognitive impairment.^38^ These findings suggest that peripheral markers may reflect central nervous system involvement in fatigue-spectrum disorders. Concurrent advances in high-throughput proteomic platforms, such as Olink and NULISA, now enable simultaneous measurement of hundreds of neurological and inflammatory analytes from minimal sample volumes, including cerebrospinal fluid and dried blood spots.^39^ If correlations can be established between these peripheral markers and central measures, e.g. PCr recovery kinetics via ^31^P-MRS and cortical lactate via MRS, they could serve as scalable proxies for neuroenergetic status in clinical trials and community settings. Validating such relationships requires concurrent sampling in well-phenotyped cohorts undergoing advanced imaging and challenge protocols.

### Research agenda: testable predictions

The MDM generates specific, testable predictions, summarized in Table 3. These predictions span neuroimaging, metabolic and physiological domains, providing a roadmap for empirical validation of the model’s four pillars.

**Table 3:** Research agenda: testable predictions derived from the MDM

Prediction	Proposed study design	Primary outcome measure
PCr recovery kinetics correlate with symptom severity	Cross-sectional or longitudinal cohort using ^31^P-MRS τPCr with standardized exertion paradigm	Association between τPCr and validated fatigue scales
Elevated cortical lactate predicts cognitive impairment	^1^H-MRS combined with standardized cognitive testing	Lactate concentration versus executive function performance
Peripheral mitochondrial dysfunction predicts post-exertional malaise	Two-day cardiopulmonary exercise testing with serial PBMC respirometry	Change in respiratory capacity versus relapse severity
NAD^+^ restoration improves clinical outcomes	Pilot randomized controlled trial of NAD^+^ precursor supplementation	Change in symptom burden with parallel biomarker shifts
Fatigue susceptibility is associated with distinct network reorganization	EEG or fMRI connectivity analysis during cognitive challenge; machine learning classification	Network efficiency metrics; classification accuracy between fatigue-susceptible and fatigue-resistant individuals.^40^

These predictions are designed to be tested in well-characterized patient cohorts using standardized challenge paradigms. Longitudinal designs incorporating repeated measures before and after physiological or cognitive stress are essential to capture the dynamic nature of the hypothesized deficits.

Recent work demonstrates that individuals with high susceptibility to mental fatigue can be identified with >95% accuracy using EEG-based functional connectivity analysis, with fatigue-resistant individuals exhibiting greater network stability and integrity.^40^ These findings suggest that baseline network properties may serve as vulnerability markers for fatigue-spectrum disorders and should be incorporated into longitudinal study designs.

### Therapeutic implications: research directions, not recommendations

If validated, the MDM would suggest potential therapeutic targets. However, current evidence does not support clinical recommendations. The following are hypothesis-driven research directions requiring investigation, with preliminary studies in related areas.^41-44^ summarized in Table 4.

**Table 4:** Hypothesis-driven therapeutic research directions

Targeted domain	Candidate intervention	Primary mechanistic research question
Energetic buffering	Creatine supplementation	Does augmentation of PCr availability improve PCr recovery kinetics (τPCr) and enhance functional capacity during cognitive or physical challenge?
NAD^+^ signaling	Nicotinamide riboside or nicotinamide mononucleotide	Does pharmacologic elevation of NAD^+^ restore recovery signaling pathways and improve symptom burden in parallel with measurable biomarker shifts?
Mitochondrial flexibility	AMPK-modulating agents (e.g. metformin)	Can enhancement of metabolic adaptability improve mitochondrial respiratory capacity and reduce relapse frequency following exertion?
Energy management	Structured pacing with heart-rate monitoring	Does physiologically guided pacing reduce the frequency and severity of post-exertional symptom exacerbation and stabilize network-level functional metrics?

These interventions are presented as hypothesis-driven research directions requiring rigorous validation in biomarker-informed clinical trials. Premature translation without mechanistic confirmation risks harm and undermines scientific credibility. Within the MDM, the four pillars may operate as a coupled nonlinear system, such that modest perturbations in buffering or metabolic flexibility could cross energetic thresholds and precipitate network-level instability.

### Broader conceptual framing: allostatic load and metabolic inflexibility

The MDM aligns with broader frameworks conceptualizing psychopathology through bioenergetic lenses. Kelley *et al.*^45^ recently proposed the Allostatic Triage Model, in which stress-induced energy scarcity drives preferential resource allocation to networks critical for short-term survival at the expense of processes supporting long-term optimization. Chronic stress may dysregulate mitochondrial and brain networks by reducing metabolic efficiency, potentially producing transdiagnostic pathological states that become entrenched through activity-dependent neuroplasticity. This perspective complements the MDM by situating neuroenergetic constraint within stress-response cascades and allostatic load, suggesting that fatigue-spectrum disorders may represent one manifestation of broader allostatic dysregulation.

Emerging evidence highlighting interactions between psychological stress and mitochondrial biology further supports integrating cellular energetics within broader stress-response models.^46^ As digital health technologies increasingly enable continuous capture of behavioural, physiological and sleep-related data, fatigue-spectrum disorders may challenge conventional monitoring paradigms by exhibiting nonlinear relapse and recovery dynamics. If neuroenergetic instability contributes to symptom variability, indirect digital signatures such as heart rate variability, activity patterns or sleep architecture may eventually serve as complementary markers of metabolic constraint. Embedding mechanistic neuroenergetic hypotheses within scalable monitoring strategies could therefore bridge molecular neuroscience with health system resilience, while remaining grounded in empirically testable biological pathways.

### Limitations and caveats

The MDM is presented as a hypothesis-generating framework, not a validated mechanistic model. Key limitations include the following:

1. Most evidence is correlational, not causal. The proposed pathways require direct testing through interventional and longitudinal studies.
2. Peripheral measures may not reflect brain energetics. Associations between blood-based biomarkers and brain function should not be assumed; concurrent validation with central measures is essential.
3. The four pillars are interrelated and may not operate independently. Their relative contributions and potential interactions remain unknown.
4. Individual variability is substantial. The model may apply to subsets of patients rather than uniformly across the fatigue-spectrum population.
5. The framework requires validation through longitudinal studies combining in vivo spectroscopy, network-level imaging and physiological challenge paradigms.
6. Causal ordering among the proposed mechanisms is not established. Whether bioenergetic deficits are primary drivers or secondary consequences of other pathophysiological processes remains to be determined.
7. Reverse causation and lifestyle confounding must be rigorously evaluated in future research, as physical inactivity, sleep disruption and dietary factors may both contribute to and result from fatigue symptoms.
8. The model does not account for potential sex differences in neuroenergetics or fatigue presentation.
9. Most cited studies have small sample sizes and lack independent replication.
10. Publication bias may overrepresent positive findings.
11. The MDM is not a clinical diagnostic tool and should not be used as such.
12. This is a hypothesis article; empirical validation is required before any clinical application.

## Conclusion

The MDM offers a unifying conceptual framework for understanding fatigue-spectrum disorders through the lens of neuroenergetic constraint. By linking PCr buffering dynamics, astrocyte–neuron metabolic coupling, mitochondrial flexibility and NAD^+^-dependent recovery signalling, the model provides a coherent architecture for interpreting emerging findings across ME/CFS, long COVID and stress-related fatigue conditions. Rather than asserting established causal mechanisms, the MDM generates mechanistically grounded, testable predictions that can guide multimodal investigation integrating spectroscopy, network-level imaging, physiological challenge paradigms and peripheral biomarker assessment.

The framework conceptualizes cumulative metabolic dysregulation as progressive bioenergetic burden that disrupts neurovascular coupling, mitochondrial efficiency and large-scale network stability. By incorporating concepts of hub vulnerability, graded energetic burden and allostatic load, the MDM situates fatigue-spectrum disorders within a broader systems neuroscience context that extends beyond categorical diagnostic boundaries.

If supported by future research, the MDM could position neuroenergetics as a central organizing principle in the neuroscience of fatigue, clarifying mechanisms, informing biomarker development and guiding rational intervention strategies across post-viral, stress-related and neuropsychiatric conditions. Its translational relevance extends to neurological disorders where fatigue is a major determinant of quality of life, including multiple sclerosis, Parkinson’s disease and traumatic brain injury. By providing a testable framework for understanding how metabolic constraints shape neural function, the MDM offers a foundation for future investigation into the mechanisms of fatigue across the spectrum of neurological and psychiatric illness.

## Supplementary Material

fcag359_Supplementary_Data
