# Supplementary material for: The metabolic debt model: cumulative bioenergetic burden and network vulnerability in brain disorders—a hypothesis article
Source: Brain Commun. 2026 Oct 16;8(5):fcag359. doi: 10.1093/braincomms/fcag359 (PMC13673110; doi:10.1093/braincomms/fcag359)
Supplement: Supplementary file 1 [file fcag359_supplementary_data.pdf]

Supplementary Materials

Glossary of Key Terms

Allostatic load: Cumulative wear and tear on biological systems resulting from chronic exposure to stress, reflecting the cost of adaptation to environmental demands.

Metabolic flexibility: The capacity of cells or organisms to dynamically adjust substrate utilization (e.g., glucose, fatty acids, ketones) and oxidative capacity in response to changing energetic demands.

Glymphatic clearance: A brain-wide perivascular pathway that facilitates cerebrospinal fluid flow through the brain parenchyma, enabling removal of metabolic waste products including lactate, adenosine, and protein aggregates.

Hub vulnerability: The principle that high-degree network nodes, brain regions with many connections, such as default mode network hubs and prefrontal cortex have elevated metabolic demands and may be preferentially susceptible to energetic constraint.

Neuroenergetic constraint: A state in which energy supply ATP availability and transfer limits neural computation, network stability, and recovery capacity.

Post-exertional malaise (PEM): Worsening of symptoms following physical, cognitive, or emotional exertion that typically occurs hours to days after the triggering activity and requires prolonged recovery.

NAD+ depletion: Reduced levels of nicotinamide adenine dinucleotide (oxidized form), a critical coenzyme for cellular energy metabolism, sirtuin-mediated signaling, and DNA repair.

Phosphocreatine (PCr) recovery kinetics (τPCr): The time constant describing the rate at which phosphocreatine stores are replenished following high-energy demand, measured using 31P-MRS.
